# Does Computer Survey Technology Improve Reports on Alcohol and Illicit Drug Use in the General Population? A Comparison Between Two Surveys with Different Data Collection Modes In France

**DOI:** 10.1371/journal.pone.0085810

**Published:** 2014-01-22

**Authors:** François Beck, Romain Guignard, Stéphane Legleye

**Affiliations:** 1 Institut National de Prévention et d'Education à la Santé (INPES), Saint-Denis, France; 2 Cermes3 - Cesames team (Research Centre Medicine, Sciences, Health, Mental Health, Health Policy), CNRS UMR 8211, Inserm U988, University of Paris Descartes, Sorbonne Paris Cité, EHESS, Paris, France; 3 Institut National des Etudes Démographiques (Ined), Paris, France; 4 Inserm, U669; Univ Paris-Sud and Univ Paris Descartes, UMR-S0669, Paris, France; The National Institute for Health Innovation, New Zealand

## Abstract

**Background:**

Previous studies have shown that survey methodology can greatly influence prevalence estimates for alcohol and illicit drug use. The aim of this article is to assess the effect of data collection modes on alcohol misuse and drug use reports by comparing national estimates from computer-assisted telephone interviews (CATI) and audio-computer-assisted self interviews (A-CASI).

**Methods:**

Design: Two national representative surveys conducted in 2005 in France by CATI (n = 24,674) and A-CASI (n = 8,111).

Participants: French-speaking individuals aged [18]–[64] years old.

Measurements: Alcohol misuse according to the CAGE test, cannabis use (lifetime, last year, 10+ in last month) and experimentation with cocaine, LSD, heroin, amphetamines, ecstasy, were measured with the same questions and wordings in the two surveys.

Multivariate logistic regressions controlling for sociodemographic characteristics (age, educational level, marital status and professional status) were performed. Analyses were conducted on the whole sample and stratified by age (18–29 and 30–44 years old) and gender. 45–64 years old data were not analysed due to limited numbers.

**Results:**

Overall national estimates were similar for 9 out of the 10 examined measures. However, after adjustment, A-CASI provided higher use for most types of illicit drugs among the youngest men (adjusted odds ratio, or OR, of 1.64 [1.08–2.49] for cocaine, 1.62 [1.10–2.38] for ecstasy, 1.99 [1.17–3.37] for LSD, 2.17 [1.07–4.43] for heroin, and 2.48 [1.41–4.35] for amphetamines), whereas use amongst women was similar in CATI and A-CASI, except for LSD in the 30–44 age group (OR = 3.60 [1.64–7.89]). Reported alcohol misuse was higher with A-CASI, for all ages and genders.

**Conclusions:**

Although differences in the results over the whole population were relatively small between the surveys, the effect of data collection mode seemed to vary according to age and gender.

## Introduction

More and more general population surveys using representative samples are attempting to assess adult drug use in European countries. In France, various surveys have been conducted since the 1990s. The National *Health Barometer* (HB) is the primary source of information about prevalence, correlates, and trends in substance use and misuse in France [1]. This survey collects data every five years from a nationally representative sample on patterns and correlates of licit and illicit drug use and related problems, with a focus on cannabis use. It is a telephone-administered interview survey. The last but one iteration of this cross-sectional survey was conducted in 2005. A few months later, the *Life Events and Health Survey* (*LEHS*) was conducted, a face-to-face interview survey mainly dealing with violence and including an audio computer-assisted self-administered section (the A-CASI) to assess licit and illicit drug use [2]. The questions in the two surveys were the same, based on the European Model Questionnaire elaborated in the late 1990s [3]. Both surveys generated prevalence estimates for lifetime substance use and past-year substance use. The aim of this paper is to compare the results of these two national surveys in order to assess the impact of the technologies used on the measurement of sensitive issues.

Although a comparison between two French surveys among adolescents in the late 1990s has already been published [4], this is the first time that such a comparison for the whole population has been possible in France, with two large samples and two different data collection methods. The availability of these two large surveys carried out during the same year with nationally representative samples presents a unique opportunity to address the issue of data collection mode effect.

A number of studies in the literature have shown how methodology can influence prevalence estimates for alcohol [5]–[8] or drugs [9], [10] in the adult population, as well as among adolescents [11]–[13]. Although it seems to be difficult to draw conclusions about areas of disagreement in comparisons of this type, most authors agree that increased reporting of substance use is a sign of improved validity in the methodology (because these behaviours tend to be underreported). When the comparison is done on data collected in the same period of time, increased reporting should be attributed to the data collection mode implemented as it cannot be a real increase [11], [14].

A large number of studies over several decades have fairly clearly underlined that self-administered questionnaires are more suitable than other data collection modes for collecting reports on sensitive behaviours [5], [9], [14]–[21]. This result is particularly convincing among adolescents and young adults [22]–[24]. Indeed, answers given to self-administered questionnaires, whether they are completed with paper and pen or by means of a laptop, seem more reliable, and are particularly well-suited to reporting behaviours that can compromise the respondent in some way, such as intimate or painful feelings or illegal behaviours [25], [26]. In particular this could be attributable to the absence of a “direct witness,” which ensures the respondent's anonymity [27]. This data collection mode also decreases the inhibitory effect of the immediate environment (in particular family, when the survey takes place within a household) [12], [13], [28]. Undoubtedly the choice of data collection mode cannot act as an absolute guarantee of quality. It requires strict compliance with a specific protocol (being alone in the room, preferably being questioned outside the home, etc.), to prevent the psychological or material environment in which the respondent is placed from undermining the advantages expected from this method.

Several studies on drug use have shown that self-administered pen-and-paper questionnaires and CASI provide similar results, both in the United States [29]–[31] or in Europe [32], [33]. However, many studies argue for computer survey technology, especially for the A-CASI in adults [8], [34] and in adolescents [11], [35] because this mode is presented as the best way to explicitly provide confidentiality and anonymity to the respondent.

In several other studies on methodology for general population surveys, the telephone interview has been also presented as a compelling alternative, as it is often considered more anonymous than face-to-face interviews with an investigator for health surveys [36]–[38], for sensitive topics [39]–[41], and especially for drug use [6], [7], [19], [42]–[47]. Phone surveys are also known to considerably reduce costs compared with face-to-face interviews.

Nevertheless, some issues have received less consideration. Our main research question was the following: Is a telephone interview equivalent to A-CASI in collecting information on drug use? From another perspective, we were also interested in measuring potential gender or age effects in these results, because these variables can more easily be taken into account than others in a multimode survey design in order to select the most appropriate data collection mode for each respondent. Methodological studies rarely receive sufficient funding to establish such thorough comparisons. But in the study of drug use, where accuracy is always questionable and where there are strong gender and age effects, these issues are nevertheless worth investigating. Age and gender differences in reported substance use prevalence rates can lead to different representations of those behaviors; these differences are probably also often linked, however, to differing propensities to declare such behaviors.

It is generally accepted that self-administration leads to more honest responses about sensitive behaviours or attitudes than interviewer administration [26]. We thus expected that the A-CASI, which is often presented as the most private data collection mode, would correspondingly encourage more frequent reporting of these behaviours. The aim of this paper is to assess the differences in responses on drug use behaviours in A-CASI and telephone surveys. To do so we compared two large random cross-sectional surveys conducted six months apart in France in 2005 and containing the same questions on drug use. We performed multivariate analyses stratified by age and gender, with special attention paid to young subjects (18–29 years old), among whom illicit substance use is most prevalent.

Because there are several differences between HB and LEHS in sampling procedures, sampling frame, interviewers and questionnaire, such a comparison can not be stricto sensu considered as an experimental design. These differences are possible sources of variation between HB and LEHS results. However, several researchers were implicated in the preparation of both surveys, and this methodological project was designed prior to the surveys. Although everything was done in the survey design to favour reliability of this comparison, it has not been possible to definitely untangle all these different effects. These points are discussed. Nevertheless, a number of important studies on data collection mode effects have been conducted following the same procedure which enables to benefit from very large sample sizes compared to experimental designs [4], [7], [8], [10], [12], [13].

## Methods

### Ethics statement

All the data were analyzed anonymously. Participants provided their verbal informed consent to participate in the study at the beginning of the questionnaire. Interviewers keyboarded the participant's consent as part of the computer-assisted interviews. For participants under 18, verbal consent was obtained from the head of the family in the same way. All procedures underwent ethical review by the appropriate national agency, the National commission for data processing and private freedoms (CNIL) and were approved. The CNIL considered that written consent was not requested for such surveys.

### Data collection

Both surveys used a household-based two-stage sampling procedure (household and individual selected using the next birthday method) and the final sample was large: 9,953 individuals aged [18–75] (among which 8,111 aged [18]–[64]) were interviewed by A-CASI and 30,514 aged [12–75] (among which 24,674 aged [18]–[64]) were interviewed by CATI. A letter was sent prior to the survey to improve response rates. Given the questionnaire was long (45 minutes on average for the CATI and 1 hour for the A-CASI) and sometimes sensitive, the interviewers had received specific training. The illicit substance use part of the questionnaires was restricted to respondents aged [18]–[64].

The *Health Barometer* (HB) was collected by a private firm on behalf of the National Institute for Prevention and Health Education (Inpes), a public establishment created in 2002 whose mission is to implement prevention and health education policies. The HB is a repeated cross-sectional telephone survey (CATI) representative of the non-institutionalized, civilian population of France. Data were collected between November 2004 and February 2005. To ensure representativeness, a subsample of 3.842 individuals with only a cell phone were added to the 26.672 individuals possessing a land-line in their homes. For this cell phone sample, the letter was not sent before the call but its sending was proposed by the interviewer. The participation rate was 65%. The data were weighted according to the number of telephone lines and the number of individuals in the household to compensate for the greater probability of being selected in households with several telephone lines and the lesser probability in households made up of many individuals. Data were then adjusted to match the French population structure in terms of gender, age group, region, town size and education, as derived from the 2005 French census, by using a calibration procedure issued from a general regression (GREG) carried out with R software. Precise descriptions of sampling and other survey procedures are available elsewhere [1]. The final sample used here comprised 24.674 persons aged 18–64.

The *Life events and health survey* (LEHS) was collected by National Institute of Statistics and Economic Studies (INSEE) interviewers for the Research, Studies, Evaluation and Statistics Directorate (DREES) of the French Ministry of Health and Sport. It was a face-to-face survey mainly exploring health and difficult life events. This cross-sectional survey is representative of the non-institutionalised, civilian population of France, aged 18–75, based on the most recent national census. Data were collected from September 2005 to November 2005. The participation rate was 72%. The most sensitive questions (sexual behaviour, alcohol and drug use) were asked via A-CASI. The respondents' privacy was ensured as the interviewer, who was alone with the respondent, left the room for the A-CASI part of the questionnaire. Out of 9,953 people interviewed face-to-face, 9,538 (95.8%) agreed to complete the interview with the A-CASI. Data were also adjusted to the French population structure as obtained from the “Continuous employment survey” carried out in 2005, both for the whole sample and for respondents who answered the A-CASI questionnaire. Precise descriptions of sampling and other survey procedures are available elsewhere [2].

Both HB and LEHS surveys rely on complex sampling: two stages of simple random sampling (SRS) for HB (phone number and then individual), stratified and three stage sampling for LEHS (stratification according to the size and socio-economic status of the area of residence, urban area for the first stage, household for the second stage then individual for the third stage). Both surveys aimed to interview the same population, but with different sampling strategies and each individual could be interviewed for both surveys, although this may be very rare (less then 0.001%). The two datasets were pooled together in order to estimate adjusted differences of A-CASI (LEHS) vs CATI (HB) in multivariate logistic regressions. The sampling design (stratification and clustering in LEHS) and the sampling weights were taken into account in the analyses, by considering HB as a separate stratum containing as many clusters as individuals.

### Measures

The two surveys employed similar assessments to estimate prevalence for lifetime alcohol and drug use, problematic alcohol use, and 12-month and 30-day cannabis use [3]. The CAGE test was used to assess problematic alcohol use. This test aims to measure the risk of alcohol dependency. It is made up of four simple binary questions [48]:

- Have you ever felt you needed to Cut down on your drinking?

- Have people Annoyed you by criticizing your drinking?

- Have you ever felt bad or Guilty about drinking?

- Have you ever felt you needed a drink first thing in the morning (Eye-opener) to steady your nerves or to feel better?

People with two or more positive answers have a high probability of being excessive alcohol drinkers or alcohol-dependent.

### Analyses

Bivariate analyses with qualitative variables were performed using design-based Pearson chi-squared tests, for the whole sample and by gender. Logistic regressions controlled for age, gender, marital and employment status, and educational level and were used to compute adjusted odds ratios (aOR) by data collection mode for a large set of substance use indicators. Analyses were first performed on the whole sample (18–64 years old), and then stratified by gender and age group: 18–29 and 30–44 years old, using SAS V9.3.2 and Stata V10.1. This strategy makes it possible, first, to take into account the strong age- and gender-related variations in psychoactive substance use [49] and second, to avoid for possible interactions between these variables and the data collection mode. Data from respondents aged 45–64 were not analysed because of very low illicit drug use prevalences.

Differences were considered significant at the 0.05 level and 95% confidence intervals were computed.

## Results

Table 1 shows that several differences between respondents to the CATI survey and respondents to the A-CASI survey remained even after weighting. There were no differences in gender and age structure between the two samples. However, with respect to level of education, in the A-CASI there were slightly fewer individuals with less than the *Baccalauréat* (final secondary school diploma) among 18- to 64-year-olds (33% versus 36% on the CATI), although this variable was part of the weighting process. This may be explained by differences in survey and weighting methods, the weighting process having been performed on the whole sample in each case (15–75 years old in HB, 18–75 years old in LEHS). Subjects interviewed in the LEHS were more likely to be employed and to live with a partner.

**Table 1:** Sample demographic characteristics for the 18–64 age group^1^.

	A-CASI (n = 8,111)		CATI (n = 24,674)		
	raw n	%	raw n	%	p-value
**Gender**					
Male	3,528	49.1	10,713	49.7	
Female	4,583	50.9	13,961	50.3	0.487
**Age**					
18–24 years	859	14.0	3,457	14.5	
25–34 years	1,646	22.2	6,193	21.5	
35–44 years	2,022	22.9	5,593	23.2	
45–54 years	1,931	22.8	4,916	22.4	
55–64 years	1,653	18.1	4,515	18.4	0.796
**Educational level**					
No diploma/CEP	1,456	23.0	3,239	22.9	
Less than Baccalauréat	2,939	32.7	8,508	35.9	
Baccalauréat	1,378	18.4	4,788	17.8	
Some higher education	850	12.5	3,345	11.3	
Higher education diploma	1,423	13.3	4,794	12.1	0.003
**Employment status**					
Employed	5,364	66.2	15,327	60.7	
Unemployed	715	9.0	2,579	11.6	
Student, retired, home maker	2,032	24.7	6,768	27.7	<0.001
**Living with a partner**					
No	2,987	27.9	9,682	32.4	
Yes	5,124	72.1	14,992	67.6	<0.001

Percentages are weighted.

p-values according to design-based Pearson chi2 test.

1 Health Barometer data are adjusted for 2005 population structure in terms of gender, age, region, agglomeration size and educational level. Life Events and Health Survey data are adjusted for 2005 population structure.

With regard to substance use prevalences, the most significant differences were with respect to alcohol-related behaviours (Table 2, Table 3 and Table 4), both for the samples overall and by gender. Among A-CASI respondents who reported lifetime alcohol use, 15.4% were CAGE-positive, whereas this was the case for only 10.8% of CATI respondents. Among the four CAGE questions, the main difference was in responses relating to the need to cut down on alcohol use: 20.4% responded positively to this question when it was posed via A-CASI, vs. only 13.7% when it was asked by CATI. Concerning illicit drugs, the differences were particularly small and never significant, whether taken separately or as a whole.

**Table 2:** Comparison between A-CASI and CATI.

	Total (n = 32,785)			Men (n = 14,241)			Women (n = 18,544)		
	A-CASI	CATI		A-CASI	CATI		A-CASI	CATI	
	(n = 8,111)	(n = 24,674)		(n = 3,528)	(n = 10,713)		(n = 4,583)	(n = 13,961)	
	% (CI)	% (CI)	p-value	% (CI)	% (CI)	p-value	% (CI)	% (CI)	p-value
CAGE positive (2+ items) (among alcohol drinkers)	15.4 [14.2–16.6]	10.8 [10.1–11.6]	<0.001	22.9 [21.0–24.8]	15.7 [14.4–16.9]	<0.001	7.7 [6.5–8.9]	5.8 [5.0–6.6]	0.006
Need to cut down on alcohol consumption	20.4 [19.1–21.7]	13.7 [12.9–14.6]	<0.001	28.3 [26.1–30.5]	19.1 [17.7–20.4]	<0.001	12.3 [10.9–13.7]	8.2 [7.3–9.1]	<0.001
People annoyed you by criticizing your drinking	12.5 [11.5–13.5]	7.9 [7.3–8.6]	<0.001	19.5 [17.8–21.2]	11.8 [10.6–12.9]	<0.001	5.3 [4.4–6.2]	3.9 [3.3–4.6]	0.018
Feel bad or guilty about your drinking	18.6 [17.4–19.8]	14.9 [14.0–15.7]	<0.001	26.8 [24.8–28.8]	20.9 [19.6–22.3]	<0.001	10.2 [8.8–11.5]	8.6 [7.8–9.5]	0.052
Have a drink early in the morning tosteady your nerves or to get rid of a hangover	1.4 [1.1–1.8]	1.2 [0.9–1.4]	0.179	1.9 [1.3–2.4]	1.7 [1.2–2.2]	0.708	1.0 [0.6–1.5]	0.6 [0.3–0.8]	0.043
Lifetime use of cannabis (at least once in lifetime)	25.4 [23.8–26.9]	25.4 [24.8–26.1]	0.973	31.3 [29.0–33.6]	33.0 [31.9–34.0]	0.196	19.7 [18.0–21.5]	18.0 [17.2–18.7]	0.062
Current use of cannabis (at least once in last 12 months)	7.4 [6.5–8.3]	7.6 [7.2–8.0]	0.710	10.6 [8.9–12.2]	11.0 [10.2–11.7]	0.668	4.4 [3.5–5.2]	4.3 [3.9–4.6]	0.837
Regular use of cannabis (at least 10 times in last 30 days)	2.5 [2.0–3.0]	2.1 [1.9–2.3]	0.131	4.0 [3.1–4.9]	3.4 [2.9–3.8]	0.195	1.1 [0.6–1.5]	0.9 [0.7–1.1]	0.365
Cocaine (at least once in lifetime)	2.5 [1.9–3.1]	2.5 [2.3–2.8]	0.899	3.7 [2.8–4.7]	3.8 [3.4–4.2]	0.973	1.3 [0.7–1.9]	1.3 [1.1–1.5]	0.939
Amphetamines (at least once in lifetime)	1.2 [0.9–1.6]	1.4 [1.2–1.5]	0.507	1.8 [1.2–2.5]	1.8 [1.5–2.0]	0.831	0.6 [0.3–1.0]	1.0 [0.8–1.1]	0.171
Ecstasy (at least once in lifetime)	2.3 [1.8–2.8]	2.0 [1.8–2.2]	0.276	3.4 [2.5–4.4]	3.0 [2.6–3.4]	0.344	1.2 [0.7–1.7]	1.0 [0.8–1.2]	0.498
Heroin (at least once in lifetime)	1.1 [0.7–1.4]	0.9 [0.8–1.0]	0.364	1.5 [0.9–2.1]	1.4 [1.1–1.6]	0.565	0.6 [0.2–1.0]	0.5 [0.3–0.6]	0.396
LSD (at least once in lifetime)	1.6 [1.2–2.1]	1.5 [1.3–1.7]	0.696	2.3 [1.6–3.1]	2.5 [2.1–2.8]	0.760	0.9 [0.4–1.4]	0.6 [0.5–0.7]	0.146
Illicit drug other than cannabis (at least once in lifetime)	4.1 [3.4–4.8]	4.5 [4.2–4.8]	0.343	5.8 [4.6–7.0]	6.4 [5.8–6.9]	0.400	2.5 [1.7–3.3]	2.7 [2.4–3.0]	0.754

Percentages are weighted.

p-values according to design-based Pearson chi2 test.

CI: confidence interval.

**Table 3:** Comparison between A-CASI and CATI among 18–29 years-old.

	Total (n = 7,874)			Men (n = 3,524)			Women (n = 4,350)		
	A-CASI	CATI		A-CASI	CATI		A-CASI	CATI	
	(n = 1,569)	(n = 6,305)		(n = 675)	(n = 2,849)		(n = 894)	(n = 3,456)	
	% (CI)	% (CI)	p-value	% (CI)	% (CI)	p-value	% (CI)	% (CI)	p-value
CAGE positive (2+ items) (among alcohol drinkers)	14.4 [11.9–17.0]	10.4 [8.9–12.0]	0.005	20.6 [16.4–24.8]	14.9 [12.5–17.4]	0.017	8.3 [5.6–11]	5.6 [4.0–7.3]	0.087
Need to cut down on alcohol consumption	16.4 [13.7–19.2]	13.3 [11.6–14.9]	0.047	22.2 [17.8–26.6]	18.1 [15.5–20.7]	0.101	10.6 [7.5–13.7]	8.1 [6.1–10.0]	0.156
People annoyed you by criticizing your drinking	12.5 [10.1–14.8]	7.5 [6.3–8.8]	<0.001	18.8 [14.9–22.8]	11.2 [9.1–13.4]	<0.001	6.1 [3.7–8.4]	3.5 [2.4–4.6]	0.029
Feel bad or guilty about your drinking	17.1 [14.5–19.7]	14.5 [12.8–16.2]	0.095	24.1 [19.7–28.5]	19.2 [16.5–21.8]	0.052	10.0 [7.1–13]	9.4 [7.5–11.4]	0.754
Have a drink early in the morning to steady your nerves or to get rid of a hangover	0.6 [0.2–1.0]	0.8 [0.3–1.3]	0.529	0.7 [0.1–1.3]	1.4 [0.5–2.2]	0.234	0.5 [0.0–1.0]	0.2 [0.0–0.5]	0.299
Lifetime use of cannabis (at least once in lifetime)	48.8 [45.0–52.6]	44.7 [43.2–46.2]	0.052	58.4 [53.3–63.5]	54.4 [52.1–56.7]	0.170	39.1 [34.1–44.0]	34.7 [32.9–36.6]	0.100
Current use of cannabis (at least once in last 12 months)	20.9 [18.0–23.8]	19.7 [18.4–20.9]	0.439	29.4 [24.8–33.9]	27.1 [25.1–29.2]	0.375	12.3 [9.3–15.3]	12.0 [10.7–13.2]	0.846
Regular use of cannabis (at least 10 times in last 30 days)	7.2 [5.4–8.9]	6.1 [5.3–6.9]	0.261	11.4 [8.3–14.6]	9.3 [8.0–10.7]	0.196	2.8 [1.4–4.2]	2.8 [2.1–3.5]	0.996
Cocaine (at least once in lifetime)	5.1 [3.5–6.7]	3.7 [3.1–4.2]	0.068	7.8 [5.1–10.4]	5.3 [4.4–6.3]	0.059	2.4 [0.9–3.9]	2.0 [1.5–2.4]	0.567
Amphetamines (at least once in lifetime)	2.7 [1.5–3.8]	1.3 [1.0–1.6]	0.003	4.3 [2.3–6.3]	1.9 [1.3–2.5]	0.004	1.0 [0.1–2.0]	0.7 [0.3–1.0]	0.372
Ecstasy (at least once in lifetime)	6.6 [4.7–8.4]	4.8 [4.1–5.4]	0.045	10.0 [6.9–13.2]	6.7 [5.5–7.9]	0.029	3.1 [1.4–4.7]	2.8 [2.1–3.4]	0.736
Heroin (at least once in lifetime)	1.6 [0.7–2.6]	1.1 [0.7–1.4]	0.181	2.7 [1.0–4.4]	1.5 [1.0–2.0]	0.109	0.6 [0.0–1.5]	0.6 [0.3–0.9]	0.994
LSD (at least once in lifetime)	2.8 [1.6–3.9]	1.9 [1.5–2.3]	0.125	5.1 [2.9–7.3]	3.0 [2.2–3.8]	0.034	0.4 [0.0–1.0]	0.8 [0.4–1.2]	0.361
Illicit drug other than cannabis (at least once in lifetime)	8.9 [6.8–11]	6.6 [5.9–7.4]	0.029	13.8 [10.2–17.5]	9.3 [8.0–10.7]	0.011	3.9 [2.1–5.8]	3.8 [3.1–4.5]	0.918

Percentages are weighted.

p-values according to design-based Pearson chi2 test.

CI: confidence interval.

**Table 4:** Comparison between A-CASI and CATI among 30–44 years-old.

	Total (n = 11,896)			Men (n = 5,199)			Women (n = 6,697)		
	A-CASI	CATI		A-CASI	CATI		A-CASI	CATI	
	(n = 2,958)	(n = 8,938)		(n = 1,254)	(n = 3,945)		(n = 1,704)	(n = 4,993)	
	% (CI)	% (CI)	p-value	% (CI)	% (CI)	p-value	% (CI)	% (CI)	p-value
CAGE positive (2+ items) (among alcohol drinkers)	13.1 [11.4–14.8]	11.0 [9.8–12.3]	0.054	19.6 [16.7–22.6]	15.9 [13.8–18]	0.039	6.5 [4.7–8.2]	6.2 [4.8–7.6]	0.802
Need to cut down on alcohol consumption	17.6 [15.6–19.6]	13.6 [12.2–15.0]	<0.001	23.7 [20.4–26.9]	18.7 [16.5–21.0]	0.013	11.4 [9.1–13.7]	8.4 [6.9–10.0]	0.030
People annoyed you by criticizing your drinking	12.2 [10.5–13.9]	8.4 [7.3–9.5]	<0.001	19.1 [16.2–22.0]	12.4 [10.5–14.3]	<0.001	5.2 [3.6–6.7]	4.4 [3.2–5.7]	0.476
Feel bad or guilty about your drinking	18.4 [16.4–20.4]	15.4 [14.1–16.8]	0.015	26.2 [23.0–29.4]	22.1 [19.8–24.4]	0.038	10.4 [8.2–12.6]	8.8 [7.3–10.3]	0.224
Have a drink early in the morning to steady your nerves or to get rid of a hangover	1.0 [0.5–1.5]	0.8 [0.5–1.2]	0.586	0.7 [0.3–1.2]	1.2 [0.6–1.9]	0.199	1.3 [0.4–2.1]	0.4 [0.1–0.8]	0.035
Lifetime use of cannabis (at least once in lifetime)	28.9 [26.4–31.4]	30.6 [29.5–31.8]	0.214	36.3 [32.6–40.1]	41.6 [39.7–43.4]	0.015	22.0 [19.0–25.0]	19.9 [18.7–21.2]	0.207
Current use of cannabis (at least once in last 12 months)	5.5 [4.3–6.8]	6.8 [6.2–7.5]	0.091	7.7 [5.7–9.8]	10.3 [9.2–11.5]	0.049	3.5 [2.1–4.8]	3.4 [2.8–4.0]	0.910
Regular use of cannabis (at least 10 times in last 30 days)	2.0 [1.3–2.6]	1.6 [1.3–1.9]	0.294	2.9 [1.8–4.1]	2.7 [2.1–3.3]	0.721	1.1 [0.3–1.8]	0.5 [0.2–0.8]	0.089
Cocaine (at least once in lifetime)	3.0 [2.0–4.0]	3.7 [3.2–4.1]	0.239	4.0 [2.5–5.5]	5.5 [4.6–6.3]	0.115	2.0 [0.8–3.3]	1.9 [1.5–2.4]	0.855
Amphetamines (at least once in lifetime)	1.0 [0.5–1.6]	1.7 [1.4–2.0]	0.081	1.5 [0.6–2.4]	2.3 [1.8–2.9]	0.188	0.6 [0.1–1.2]	1.0 [0.7–1.4]	0.266
Ecstasy (at least once in lifetime)	1.8 [1.1–2.5]	2.2 [1.8–2.5]	0.386	2.3 [1.1–3.5]	3.5 [2.9–4.1]	0.119	1.3 [0.4–2.3]	0.9 [0.6–1.1]	0.297
Heroin (at least once in lifetime)	1.2 [0.6–1.9]	1.5 [1.1–1.8]	0.556	1.7 [0.6–2.8]	2.3 [1.7–2.8]	0.417	0.8 [0.1–1.5]	0.7 [0.4–1.0]	0.754
LSD (at least once in lifetime)	1.7 [0.9–2.5]	1.8 [1.4–2.1]	0.932	1.9 [0.7–3.0]	3.0 [2.4–3.7]	0.130	1.6 [0.5–2.7]	0.5 [0.3–0.7]	0.002
Illicit drug other than cannabis (at least once in lifetime)	4.0 [2.9–5.1]	5.5 [5.0–6.1]	0.026	4.8 [3.2–6.4]	8.2 [7.2–9.2]	0.002	3.2 [1.7–4.6]	2.9 [2.4–3.5]	0.743

Percentages are weighted.

p-values according to design-based Pearson chi2 test.

CI: confidence interval.

Table 5 shows that after adjustment for age, gender, marital and employment status and educational level in logistic regressions, differences in CAGE test responses remain: compared to the CATI, the A-CASI yielded more positive responses for the whole sample (aOR = 1.60 [1.38–1.77]) and for both age groups (aOR = 1.51 [1.16–2.00] in 18–29 and aOR = 1.31 [1.07–1.60] in 30–44). In the male subsample, ORs were significant for the whole sample (aOR = 1.63 [1.41–1.89]) and for both age groups (aOR = 1.48 [1.07–2.04] in 18–29 and aOR = 1.37 [1.07–1.74] in 30–44); in the female subsample, OR was significant only for the whole sample (aOR = 1.41 [1.13–1.75]) and for the 18–29 age group (aOR = 1.61 [1.02–2.55]). In addition, people more often reported a regular cannabis use on the A-CASI (aOR = 1.32 [1.04–1.67]), particularly women in the 30–44 age group (aOR = 2.56 [1.11–5.88]), while men aged 30 to 44-year-olds more often reported having ever used cannabis in their lifetime on the CATI (aOR = 0.81 [0.68–0.97]). Lifetime use of illicit drugs other than cannabis was less often reported by men in the 30–44 age group on the A-CASI (aOR = 0.61 [0.42–0.89]). People aged 18 to 29 more often reported lifetime use of illicit drugs via A-CASI (aOR = 1.45 [1.09–1.91]), escpecially men (aOR = 1.66 [1.18–2.33]). Thus, men aged 18–29 more frequently reported having used ecstasy, amphetamines, LSD, cocaine and heroin on the A-CASI questionnaire. Among women, results were close for lifetime use of illicit drugs other than cannabis, except for LSD for which they reported more on the A-CASI (aOR = 3.60 [1.64–7.89]).

**Table 5:** Multivariate logistic regressions comparing A-CASI and CATI: OR and 95%CI.

Odds ratio for A-CASI (1) vs CATI (0)		Total (n = 32,785)		Men (n = 14,241)		Women (n = 18,544)	
	Age group	OR	95%CI	OR	95%CI	OR	95%CI
CAGE positive	18–64	**1.60**	1.38–1.77	**1.63**	1.41–1.89	**1.41**	1.13–1.75
	18–29	**1.51**	1.16–2.00	**1.48**	1.07–2.04	**1.61**	1.02–2.55
	30–44	**1.31**	1.07–1.60	**1.37**	1.07–1.74	1.19	0.83–1.71
Lifetime cannabis use	18–64	0.98	0.89–1.07	0.91	0.80–1.03	1.08	0.95–1.23
	18–29	1.14	0.97–1.35	1.16	0.92–1.46	1.12	0.90–1.40
	30–44	0.93	0.81–1.06	**0.81**	0.68–0.97	1.10	0.71–1.69
Current cannabis use	18–64	1.05	0.90–1.22	1.04	0.85–1.26	1.06	0.84–1.34
	18–29	1.16	0.96–1.42	1.22	0.95–1.56	1.04	0.76–1.43
	30–44	0.89	0.68–1.16	0.80	0.58–1.10	0.97	0.72–1.31
Regular cannabis use	18–64	**1.32**	1.04–1.67	1.32	0.99–1.75	1.39	0.87–2.21
	18–29	1.13	0.90–1.41	1.36	0.95–1.93	1.15	0.63–2.10
	30–44	1.50	0.99–2.28	1.29	0.80–2.08	**2.56**	1.11–5.88
Ecstasy (lifetime)	18–64	1.20	0.93–1.54	1.21	0.89–1.65	1.17	0.75–1.84
	18–29	**1.44**	1.05–1.98	**1.62**	1.10–2.38	1.07	0.61–1.86
	30–44	0.92	0.59–1.45	0.72	0.41–1.25	1.68	0.78–3.60
Amphetamines (lifetime)	18–64	0.92	0.67–1.27	1.09	0.73–1.62	0.64	0.34–1.20
	18–29	**2.26**	1.40–3.65	**2.48**	1.41–4.35	1.77	0.71–4.41
	30–44	0.66	0.38–1.15	0.69	0.34–1.37	0.60	0.24–1.51
LSD (lifetime)	18–64	1.12	0.83–1.50	1.00	0.71–1.40	1.57	0.86–2.86
	18–29	**1.66**	1.02–2.70	**1.99**	1.17–3.37	0.59	0.13–2.63
	30–44	1.09	0.66–1.80	0.66	0.34–1.29	**3.60**	1.64–7.89
Cocaine (lifetime)	18–64	1.02	0.80–1.31	1.04	0.79–1.39	0.98	0.62–1.57
	18–29	**1.49**	1.04–2.14	**1.64**	1.08–2.49	1.17	0.59–2.29
	30–44	0.88	0.61–1.29	0.78	0.50–1.20	1.14	0.59–2.22
Heroin (lifetime)	18–64	1.30	0.90–1.87	1.25	0.81–1.94	1.44	0.71–2.89
	18–29	1.86	0.97–3.56	**2.17**	1.07–4.43	1.15	0.25–5.33
	30–44	0.97	0.55–1.72	0.84	0.41–1.69	1.41	0.53–3.73
All illicit drugs combined (except cannabis)	18–64	0.94	0.77–1.13	0.94	0.74–1.19	0.94	0.67–1.33
	18–29	**1.45**	1.09–1.91	**1.66**	1.18–2.33	1.03	0.62–1.69
	30–44	0.76	0.56–1.04	**0.61**	0.42–0.89	1.13	0.68–1.87

N.B.: Each regression is controlled for gender, age, marital and professional status and educational level.

ORs in bold are significant at 0.05 level.

The reference category for predictive variables is “No use” for illicit drugs or “Non-problematic use”, i.e. CAGE (cut-down annoyed guilty eye-opener) negative for alcohol.

Data from the 13,015 respondents aged 45–64 were not analysed because of very low illicit drug use levels.

OR: odds-ratio; CI: confidence interval.

## Discussion

Altough all representative surveys across Europe show age and gender disparities in drug use, few methodological studies have been undertaken to test the influence of data collection mode on drug prevalences reported on surveys, particularly in different age and gender groups. A major feature of this analysis was our use of two large samples from the non-institutionalized French population to compare prevalences of drug use according to data collection mode in three age groups, covering a large part of the population (18–64 years), and in both genders.

### Findings

We found that, while overall prevalences are very similar for the two data collection modes, differences are found between the different data collection modes according to the age and the gender of the respondents. Males between the ages of 18 and 29 more often responded positively on the CAGE questionnaire and more often reported lifetime use of ecstasy, amphetamines, LSD, cocaine and heroin via A-CASI than in the CATI. Females aged 30 to 44 were more likely to report regular cannabis use and lifetime LSD use via A-CASI.

According to our results, the A-CASI seems particularly suitable for young men, whereas telephone surveys seem well-suited to people aged 30 and over, particularly in men. Reporting on use of cannabis (current and regular) does not appear to be influenced by data collection mode. Differences between data collection modes were less pronounced among women, especially for lifetime use of illicit drugs other than cannabis.

### Comparison with other studies

It is generally assumed that under-reporting can be a serious problem for highly sensitive or illegal behaviours such as alcohol misuse or illicit drug use, and that underreporting can be reduced by greater privacy in the mode of survey administration [14], [19], [28], [44] and that self-administration leads to more honest responses on sensitive behaviours [26]. Our results suggest that this general pattern may be true for young males, but not valid for all age groups and gender.

A differential effect of the data collection mode by gender has already been reported in a Belgian study [33]. This comparison of self-administered pen-and-paper questionnaires and A-CASI among school children (aged [13]–[20] years old) showed that using a computer yielded higher prevalences among girls and lower prevalences among boys. These results were found in adolescents, while our results were derived from young adults. It can be noted that they are not in accordance with the results for our youngest age group (18–29 years old). This suggests that differential gender effects should be more deeply analyzed in future research.

### Interpretations of the findings

Apart from data collection mode, which is considered in the literature as a major influence on the way respondents answer sensitive questions [4]–[13], [15], several methodological differences could influence prevalence estimates for substance use. Nevertheless, the sampling procedures and the sampling weights were taken into account in the analyses, by considering HB as a separate stratum containing as many clusters as individuals.

Some of the more salient differences between the *Health Barometer* and the *LEHS* include privacy considerations (presence of the interviewer or of other people) and response rate. On one hand, the LEHS was performed face-to-face by interviewers from the French National Institute for Statistics and Economic Studies (INSEE), using computerized self-administration methods (A-CASI) to collect drug-related data. On the other hand, the HB was a telephone survey conducted by a private polling agency. It is difficult to know, however, how much the kind of interviewer influences respondents' perception of privacy and confidentiality. Effects could differ by gender and age group. For example, older people may be more mistrustful towards computers, perceiving them as more likely to enable breaches in confidentiality, or else they may have poorer computing skills. More generally, men may be more mistrustful towards a face-to-face interviewer, as they are generally less trusting about surveys and less willing to participate than women. It could also be because they are more prone to use illicit drugs than women [1], [2]. On the other side, women may be more reluctant to admit problems and deviant behaviours, particularly drug use: these are male behaviours that generally expose women to formal or informal reproaches [50]. A setting that is free from direct interaction with another person could be favourable. Our results are in accordance with these hypotheses, but additional investigations are needed to explore why the results vary according to substance.

### Limitations

Although our study is based on large representative samples using the same questions, it is subject to several limitations that are common in such methodological comparisons. The interviews were conducted by trained interviewers and response rate was satisfactory for such health surveys. However, selection bias cannot be ruled out and some populations, especially the most deprived such as homeless people, are likely to be under-represented in both surveys, even though some were interviewed thanks to the HB sample based on mobile phone numbers. However, due to the small size of such groups, this has only a small effect on population-level estimates, as it has already been shown for alcohol prevalence [51].

In general population surveys, recall bias in substance use reporting is a major concern. This is considered as a threat for survey measurements of alcohol consumption in general [52]. Indeed, survey questions encourage error because they do not help respondents to recall extensively all their drinking occasions or because respondents must answer based on standard drink sizes that often do not match their own drinking style [53]. Concerning illicit drugs, recall bias is probably less important than for alcohol consumption. In studies examining agreement between timeline follow-back for self-reported use and biological measures for illicit substances, agreement rates were considered high [54]. However, longitudinal cohort studies have suggested that re-interviews about drug use often lead to recanting resulting in a decreased reports of lifetime substance use [55].

Several of the substance use behaviors are very rare: for example, lifetime LSD and heroin uses do not exceed 1% among women. This could explain the wide 95% confidence interval obtained for lifetime LSD use odds-ratio for women aged 30–44 ([1.64–7.89]) or for lifetime heroin use odds-ratio for women aged 18–29 ([0.25–5.33]). However, this does not affect the salience of the approach nor the overall quality of the models.

Differences were considered significant at the 0.05 level and no adjustment for multiple comparisons was made. However, the present analysis is suitable for a descriptive purpose.

The few months between both surveys could be underlined as a possible factor of explanation for the differences, in particular concerning seasonal behaviours such as alcohol intake, with differences between holidays and normal working weeks, New Year's Eve parties, etc. Several studies have shown a seasonal effect in alcohol use. This effect is particularly strong for specific populations such as professional sportsmen [56] and which is mainly a “January effect” in most European countries and the Northern Hemisphere [57], [58]. But this factor probably plays a minor role because the HB fieldwork was interrupted from 20^th^ December 2004 to 10^th^ January 2005 in order to minimize both overindulgence during the holiday season and New Year's resolutions toward temperance. Moreover, except for cannabis regular use, which is calculated on the last 30 days, the indicators relied on large period of time (lifetime or last 12 months, including all seasons for all the respondents). Such indicators are much less impacted by seasonal effects than daily or recent use of alcohol or drugs.

The databases used in the two surveys (persons living in households in LEHS, members of households equipped with a landline or mobile telephone in HB) could also explain part of the differences in prevalences. However, individuals without a landline but who have a mobile phone were included in the HB sample, and households with no telephone are quite rare in France (about 1%). Moreover, a study conducted on several editions of the National Household Survey on Drug Abuse (NHSDA) found very similar levels of drug use in reachable populations (about 80,000 individuals) and among individuals with no telephone (5,800 individuals) [59]. The differences, although they varied according to the substance, were found to be always small [59].

The LEHS had a slightly higher response rate than the HB (72% vs. 65%), a factor that would generally be expected to increase substance use estimates, as difficult-to-reach respondents have been shown to have higher rates of risky behaviours [60] and substance use particularly [44], [61], [62]. Recent results show, however, that this distinction is probably negligible with respect to the effect of the mode of collection [10]. Moreover, despite efforts made to reach households (up to 20 calls before giving up a phone number), 8% were unreachable in the phone survey.

There could be also a halo effect: in other words, the influence of questions occurring earlier in the questionnaires. One study showed that having opportunities to express positive behaviours at the beginning of a survey legitimised the later reporting of practices that are difficult to admit to. In addition, the closer the theme covered by the survey was to a theme considered sensitive by public opinion (such as violence or suicide), the higher the reported levels of alcohol use [63]. The results of another study suggested that the larger the number of questions with regard to a given theme, the greater the probability of obtaining a positive answer concerning a deviant behaviour on this theme [64].

Indeed, although the questions in the drug module were designed to be identical, the questionnaires on the LEHS and the HB nevertheless slightly differed. In both surveys, the drug module was placed toward the end of the questionnaire, but the questions preceding this module were not the same, which could have intensified a possible halo effect. It is particularly difficult to untangle a factor of this kind from other possible effects, in particular those related to data collection mode. Nevertheless, there were so few differences in the design or in the wordings of the questions that this issue is unlikely to have affected drug use prevalence estimates.

## Conclusion

The results of this study are twofold: first, with slight differences in the main themes broached in the questionnaires and with different data collection modes, prevalences of illicit drug use appeared to be quite similar between surveys, unlike alcohol misuse. Second, there were marked differences according to gender and age. On one hand, computer survey technology improved reports on alcohol and drugs for the whole population, and reports on illicit drug use for young men only. On the other hand, the use of a telephone survey yielded very similar results for women for most of indicators. The methodological research discussed above demonstrated the impact of the mode of data collection on the quality of responses some time ago. The prevalences found in our study do not, however, clearly demonstrate the unilateral superiority of one mode in collecting data on sensitive topics. From the current point of view, according to which higher prevalences are a sign of more reliable reporting, the A-CASI seems to be more suitable for young men, whereas the telephone interview offers convincing results for women and people aged 30 and over. Thus, our results may support multi-mode approaches as suitable solutions to improve response quality in general population surveys, although it may lead to very complex design. Further research is thus needed to understand gender and age differences and to replicate this study. Beyond the two modes that were chosen for these surveys, other promising modes such as Telephone Audio Computer-Assisted Self-Interviewing (T-ACASI) should certainly be considered in the European setting, following recent experiments [65]–[69]. Finally, our findings underline the fact that if the computer survey technology seems to improve reports on alcohol and illicit drug use in the general population, our results also showed that CATI remains an efficient mode, sometimes probably even more convenient in elderly people or in women.
